# An Evidence-based rationale for a North American commercial bumble bee clean stock certification program

**DOI:** 10.26786/1920-7603(2023)721

**Published:** 2023-01-24

**Authors:** James P. Strange, Sheila R. Colla, Laurie Davies Adams, Michelle A. Duennes, Elaine C. Evans, Laura L. Figueroa, David M. Lehmann, Heather Moylett, Leif Richardson, Ben M. Sadd, James W. Smith, Tamara A. Smith, Amber D. Tripodi, Edward M. Spevak, David W. Inouye

**Affiliations:** 1 Department of Entomology, The Ohio State University, Columbus, OH 43214; 2 Faculty of Environmental and Urban Change, York University, Toronto, ON, M3J1P3, Canada; 3 Pollinator Partnership, 600 Montgomery, Suite 440, San Francisco, CA 94111, USA; 4 Department of Entomology, University of Minnesota, Saint Paul, MN 55108 USA; 5 Department of Environmental Conservation, University of Massachusetts, Amherst, Amherst, MA, 01003, USA; 6 Department of Entomology, Cornell University, Ithaca, NY, 14850, USA; 7 Department of Environmental Conservation, University of Massachusetts, Amherst, Amherst, MA, 01003, USA; 8 Center for Public Health and Environmental Assessment (CPHEA), Health and Environmental Effects Assessment Division, Integrated Health Assessment Branch, US - Environmental Protection Agency, Research Triangle Park, NC, 27711, USA; 9 Unaffiliated, Garner, North Carolina, 27529, USA; 10 The Xerces Society for Invertebrate Conservation, 628 NE Broadway, Suite 20, Portland, OR 97232-1324, USA; 11 School of Biological Sciences, Illinois State University, Normal, IL 61790, USA; 12 Retired USDA-Animal and Plant Health Inspection Service, Fuquay-Varina, NC 27526, USA; 13 Unaffiliated, Minneapolis, MN 55406; 14 Unaffiliated, Raleigh, North Carolina, 27604, USA; 15 Center for Native Pollinator Conservation, Saint Louis Zoo, One Government Drive, St. Louis, MO 63110, USA; 16 Department of Biology, University of Maryland, College Park, MD 20742, and Rocky Mountain Biological Laboratory, PO Box 519, Crested Butte, CO 81224, USA

**Keywords:** *Bombus*, bumble bee, management, disease, parasite, pathogen, clean stock

## Abstract

The commercial production and subsequent movement of bumble bees for pollination of agricultural field and greenhouse crops is a growing industry in North America and globally. Concerns have been raised about the impacts of pathogen spillover from managed bees to wild pollinators, including from commercial bumble bees. We recommend development of a program to mitigate disease risk in commercial bumble bee production, which will in turn reduce disease stressors on wild pollinators and other insects. We provide recommendations for the components of a clean stock program with specific best management practices for rearing commercial bumble bees including related products such as wax, pollen, and nesting material.

## Introduction

International and domestic commerce of both plant and animal species is regulated by local, national, and international laws, including for threatened and endangered species. Another category of regulation stems from attempts to prevent the spread of unwanted species such as insect pests and weeds, or parasites and predators that could affect native species. A potential problem with commerce of livestock, including bees, is the opportunity that movement of animals from one region to another creates for moving parasites and pathogens. For bumble bees, endoparasites include viruses, bacteria, Protozoa, fungi, and nemotodes (Figueroa et al. 2023), and ectoparasites or nest parasites can also be of concern for individual and colony health (Evans et al. 2023). There is the potential for many of these to be moved unintentionally as a consequence of commercial trade of their host species.

Bumble bees (*Bombus* spp.) are important pollinators of commercially grown crops, a variety of garden vegetables, and native flowering plants. Approximately 40 bumble bee species are native to the United States and Canada (Williams et al. 2014) and three of them are commercially available in those countries. By far the most economically important managed bumble bee species in the United States and Canada is *Bombus impatiens*, native to the eastern United States and Canada (Velthuis & van Doorn 2006). However, *Bombus huntii* is available for use in western Canada and *Bombus vosnesenskii* was recently approved for use in California and is now being sold commercially, and it is expected that use of these new commercial species will grow in the western USA and Canada in the future. Currently, these species are produced in facilities in Michigan (USA) and Ontario (Canada) and shipped throughout North America for crop pollination, most notably, greenhouse-grown tomatoes (Strange 2015; Velthuis & van Doorn 2006).

The dangers of introducing bumble bee species outside of their native ranges are well known now, whether they are introduced to areas where bumble bees have never occurred (e.g., Tasmania Hingston & McQuillan 1999; Stout & Goulson 2000), or where there are native species (e.g., South America Chalcoff et al. 2022; Montalva et al. 2017; Smith-Ramirez et al. 2021; Torretta et al. 2006).

While the commercial producers of bumble bees make efforts to maintain clean stock in production facilities (Huang et al. 2015), and provide guidelines to end-users for containment when bees are sold outside of their native range, commercial bumble bee hives are not always isolated from wild bumble bee communities when in use, because bees often forage outside of greenhouses via vents (Whittington et al. 2004). Bumble bees are also deployed frequently in open-field situations to augment pollination of field tomatoes, tree fruit, and berry crops. The use of these bees where they can come into contact with wild bees poses a clear risk for the movement of pathogens and parasites within and beyond the bumble bee community (Colla et al. 2006; Fürst et al. 2014; Murray et al. 2013). Managed bumble bees have the potential to amplify existing pathogens and parasites in the wild bumble bee community, through pathogen spillback (Pereira et al. 2021), but the introduction of pathogens and parasites with managed colonies represents a greater concern. High pathogen incidence has been correlated to facilities that deploy commercial bumble bee hives, leading to concerns of pathogen spillover (Colla et al. 2006; Murray et al. 2013).

Notably, declining bumble bee populations in the United States (Cameron et al. 2011) and Canada have been linked to higher levels of pathogens (Cordes et al. 2012; Kent et al. 2018). However, a clear causative link between population status and infection remains elusive, due to a lack of baseline data on differential susceptibilities among North American fauna, although in other regions differences of pathogen infection impacts among species has been documented (Rutrecht & Brown, 2009). Declines in some species have raised concerns about extinction risk and over 20% of North American species have been identified through the International Union for the Conservation of Nature Red List as ‘at risk’ of extinction (Cameron & Sadd 2020). In addition, several species are legally recognized in the United States and Canada as endangered, including *Bombus affinis*, the Rusty Patched Bumble Bee, which is federally protected in both countries. The impacts of commercial bumble bees on these declining species are poorly understood, but previous disease outbreaks in rearing facilities have been implicated in declines (Flanders et al. 2003).

Commercial bumble bee production begins in captivity when lab-raised queens are provisioned with honey bee-collected pollen and sugar syrup and confined to a nest box where they commence nesting (Huang et al. 2015; Velthuis & van Doorn 2006). Within a few days of confinement, the queen bumble bee will oviposit on the pollen mass and begin brooding her developing offspring. More pollen is provided as needed as the developing nest remains in isolation in the facility. As worker bees reach adulthood and the nest grows, the nest is moved to a shipping box (= hive) and is ready for sale about 60 days after nest initiation. Once colonies reach a desired size (*e.g*., 50–100 workers), the nests are shipped from the production facilities to growers, and do not return (Huang et al. 2015), nor are the nesting materials from sold colonies returned to the facility, however a percentage of the colonies reared in facilities must be retained to supply future reproductive individuals for the operation (Huang et al. 2015; Velthuis & van Doorn 2006). Growers dispose of the colonies once their crop has completed flowering or the colony starts producing reproductive individuals instead of workers.

Although the bumble bee production environment is closed, the rearing system does have external inputs. Notably, sugar and pollen must be supplied to developing colonies, and nesting material is also essential (Huang et al. 2015). Nesting boxes from major bumble bee producers are plastic boxes that are manufactured for the purpose of rearing bees. These boxes are sterile when introduced into the production system.

Similarly, sugar syrup is provided, generally in a proprietary nutrient and preservative mixture, and this is sterilized before delivery (Velthuis & van Doorn 2006). Pollen must be obtained in large quantities for commercial production and this necessitates purchasing bulk pollen that has been collected by honey bee keepers from honey bee hives (Velthuis & van Doorn 2006). Pollen from honey bee hives is collected using standard pollen traps deployed on the entrance of a honey bee colony. The traps remove pollen from the corbiculae of returning honey bee foragers and collect this pollen in trays that the beekeeper can empty. Because the pollen is retrieved from a biological system and has had contact with honey bees in a hive, it is frequently contaminated with pathogens (Chen et al. 2006; Gilliam et al. 1988; Graystock et al. 2013a; Graystock et al. 2013b; Higes et al. 2008) and detritus, and may be contaminated with pesticides or other environmental contaminants (Mullin et al. 2010). Pollen sourcing thus represents a significant risk to bumble bee production. It is unknown to what extent new queens or male bumble bees are brought into rearing facilities to increase genetic diversity and avoid inbreeding of captive stock, but that is another possible external input.

A large number of pathogens and parasites are known to attack and infect bumble bees (Goulson 2010); however, not all of the pathogens pose risks on an economically important scale (reviewed in Evans et al. 2023; Figueroa et al. 2023). Likewise, some parasites that are already abundant in the wild would seem to pose little threat of being spread by captive reared bees, due to their complex life cycles. Furthermore, some pests such as wax moths or Indian meal moths can become a problem in rearing facilities, but probably pose little threat to bees in native communities. Yet, some pathogens such as *Vairimorpha* (*Nosema*) *bombi*, *Apicystis bombi*, *Crithidia bombi*, a variety of viral diseases, and potentially emergent pathogens can infect commercial colonies and be moved quickly through shipments across the continent. Bee movement regulation and clean stock guidelines are needed to ensure tolerable levels of pathogens, parasites, and pests, are not exceeded and so that outbreaks are quickly detected and contained. Implementation of a clean-stock program would align with needs identified in the U. S. National Strategy on Pollinators in the Pollinator Research Action Plan (2015) and the National Strategy for Biosurveillance (2012), both of which highlight the need for detection and monitoring of diseases with potential to impact agricultural production. A clean-stock certification program would help reduce the threat and impacts that managed bumble bees have on wild bee populations, and help commercial companies avoid economic costs associated with outbreaks.

In this document we adopt the term “potentially deleterious symbiont” to mean organisms (including viruses) that have a known or suspected deleterious association with bumble bees in captivity or the wild. Not all symbionts are thought to have ecologically or economically relevant impacts. We define “clean-stock program” as a documented system 1) to detect pathogens and parasites of concern in commercial rearing facilities that pose a threat to wild bees, 2) to prevent the spread of infections both within and outside of facilities, and 3) to produce actionable information for federal, state, and provincial regulators and conservation professionals if a suspected disease outbreak occurs. The clean-stock program can equally apply to laboratories rearing bumble bees for research or conservation purposes.

In previous papers we have 1) summarized what is known about potentially deleterious endosymbionts (Figueroa et al. 2023), and 2) summarized what is known about potentially deleterious ectosymbionts (Evans et al. 2023). In this paper we provide recommendations for a clean stock program for commercial bumble bees including related products such as wax and pollen. The development of a clean-stock program would enable producers, regulators, conservation groups, and end users of bumble bees to ensure that all reasonable measures are being taken to maintain healthy bumble bee communities in both production and wild systems.

## The Clean Stock Program

### Uncertainty and known risks

In many cases, the effects of parasites on individual and colony health, stability, and growth are unknown or at best, only partly known. Often, negative effects of these organisms at the colony level will only become apparent when colonies or individuals are experiencing other stressors (Brown et al. 2003). Because much of the experimental work documenting the effects of parasite infection has been in single species (largely either *B. impatiens* or *B. terrestris*), it is unclear how pathogenicity in one host translates to other species across the genus (Cameron & Sadd 2020). Because there is little support in the literature for a safe level of most parasite-host systems, we recommend that commercial bumble bee producers voluntarily adopt clean stock procedures to isolate and screen all colonies with symptoms of pathogen and parasite infections and take reasonable effort to determine the causal agents of symptoms. Colonies exhibiting symptoms should never be shipped for commercial sale. Moreover, we recommend regular testing for different pathogens and parasites to pinpoint any infections before symptoms appear and spread within the rearing facility, and before colonies are shipped for commercial operation where the managed bees could contaminate shared flowers leading to spillover into of wild bee communities.

Our understanding of pathology varies by parasite group, as do the screening techniques available. Virus levels that cause pathology remain largely unknown for bumble bees. For example, deformed wing virus has been found in bumble bees numerous times when no pathology is clearly evident (Dolezal et al. 2016; Gusachenko et al. 2020; Levitt et al. 2013; Li et al. 2011; McMahon et al. 2015; Singh et al. 2010). Likewise black queen cell virus seems to be widely distributed among bumble bee species (Peng et al. 2011; Radzevičiūtė et al. 2017; Reynaldi et al. 2013; Sachman-Ruiz et al. 2015; Singh et al. 2010), but a specific pathology in commercial or research colonies of bumble bees is unknown. Complete elimination of viruses in rearing facilities is unlikely; however, reduction of viral load for all known viruses is important to produce disease-free bees. Pollen used in the rearing process, thus, should be brought in virus-free or sterilized appropriately before bees are exposed to it (Simone-Finstrom et al. 2018).

We recognize that large-scale commercial rearing of biological organisms for agricultural use comes with a variety of risks, both known and unknown. Risk mitigation is most successful when risks are enumerated prior to appearing and managed. However, not all risks to commercial bumble bee production can be known *a priori* and there are known knowns, known unknowns and unknown unknowns. For example, producers know several management techniques to ensure year-round production of bumble bees for commercial pollination service (e.g., Röseler 1985). The research and conservation community knows which species are used, where they can be shipped (there are some state-level restrictions in the USA), and several of the management strategies employed. We also know that we do not know other proprietary business and management strategies that these companies employ, such as the number of colonies that are shipped annually, nor sanitation and sterilization strategies for bee feed, nesting material, and equipment. Further, there are likely issues related to health and sanitation of which we are unaware, particularly for emergent diseases. For example, there is no public reporting of disease outbreaks in rearing facilities that researchers can access. The protection of the proprietary nature of these production processes places wild pollinators and crop producers at some risk and the historical reluctance of commercial bumble bee producers to share this information may in fact be creating concern in the conservation, regulatory, and scientific communities where it is not needed. Thus, channels for communication among producers, consumers, conservationists, regulators, and scientists should be cultivated so that commerce can proceed and the health of wild populations of bees can be ensured.

Known risks to wild and managed pollinators include the escape of managed bumble bee species from containment and pathogen spread to wild bee communities. It is now well known and documented that commercial bumble bees escape from containment and establish in new regions (Matsumura et al. 2004; Morales et al. 2013; Roig Alsina & Aizen 1996) including in North America (Looney et al. 2019; Ratti & Colla 2010) and South America (Aizen et al. 2019). Although it might seem that screening all openings in greenhouses would be effective in reducing escape, this measure has apparently not been adopted rigorously, or is subject to a significant failure rate. Avoiding or minimizing use of commercial colonies in crops outside of greenhouses could also reduce transmission of parasites and diseases to native populations.

The degree to which alien commercial bees outcompete conspecifics is not yet fully understood (Ings et al. 2006), but concerns exist (Aizen et al. 2019), and evidence suggests that genetic introgression (Kondo et al. 2009) and transport of parasites and spillover onto wild bees can occur (Alger et al. 2019; Colla et al. 2006; Goka et al. 2001; Maharramov et al. 2013; Purkiss & Lach 2019; Schmid-Hempel et al. 2014). Despite knowing that these risks exist, the degree to which they might impact agriculture in various parts of the world with differing climates, cultural practices, and agricultural systems is unknown. Further, while we have some knowledge of the impacts that commercial bumble bees have on native bumble bees, there is little knowledge of the impacts these bees might have on honey bees or other managed and wild pollinators. Ensuring both implementation of clean stock protocols and access to production and sales records would lessen the degree of uncertainty in this system and would allow for robust contact tracing and containment should releases or disease outbreaks occur.

A clean stock certification program would decrease the levels of uncertainty that exist around commercial bumble bee health. To address these issues and mitigate risk to native bees and commercial honey bee and bumble bee pollinators, a strong commitment by commercial producers to broader pollinator health is needed. A voluntary and transparent clean stock program that emphasizes the common interests of commercial producers and the pollinator conservation community would address many of the concerns surrounding bumble bee health. For example, processes of pollen sterilization could be made available, sanitation processes could be published, records of shipments could be made public, and a culture of openness should be cultivated in areas of business operations that impact the community health of bees. Voluntary annual inspection of rearing facilities by a clean stock certification group would also accomplish the goals for transparency, disease suppression, and wild bee conservation. Industry standards developed for vertebrate livestock such as the Animal Disease Traceability General Standards (Animal and Plant Health Inspection Service 2019) and the NLRAD System Standards (Animal and Plant Health Inspection Service 2020) could serve as models for the industry and conservation and scientific partners to construct a clean stock certification and tracking program.

### Clean stock program components

A clean stock program to ensure healthy colonies are available to support agricultural needs, while simultaneously protecting wild and managed bee populations, should include the following Best Management Practices:

1. Screening and detection
2. Quarantine and isolation
3. Sanitation and prevention
4. Treatment
5. Forensic (tracing) capacity

The following controls are critical processes for the success of a clean stock program to prevent the spread of disease within facilities and to prevent spillover of disease-causing agents to wild bee communities. Producers seeking certification should maintain a written, publicly accessible protocol of processes related to production of clean stock, including a strong commitment to transparency in production processes. Employees should receive annual training in disease prevention and containment. Companies seeking certification should have an annual review and/or inspection of facilities to ensure compliance.

### Screening and Detection

Screening for disease in rearing facilities would ideally involve a two-tiered system that is integrated into a system of quarantine and isolation. The first tier is visual inspection of colonies throughout the rearing facility that is aimed at detecting symptomatic infections. The second tier is one of testing asymptomatic colonies to detect latent disease spread before symptoms appear. The first tier would involve all colonies that are maintained in the production facility and would occur regularly when colonies are fed, moved from starter to full colony boxes, and before shipment or transfer to gyne production lines. Visual screening for symptoms would include, but not be limited to, looking for lethargic bees, trembling or shivering, deformed wings or legs, unusual patterns of defecation and odd odors, failure to thrive, ejected larvae, and other unusual behavior. Identification of symptoms of infection in the first tier should be followed up using appropriate visual or molecular approaches, as outlined previously (Evans et al. 2023; Figueroa et al. 2023), to verify causative agents.

The second tier of testing would employ random testing to detect the presence of pathogens that are not yet inducing symptoms. The exact program for testing could vary, but at a minimum should include testing a random subset of 20% of all colonies in a rearing facility (Huang et al. 2015) using non-specific Trypanosomatid (*e.g., Crithidia* spp.) and Microsporidian (*e.g., Vairimorpha* spp.) PCR primers, as well as primers specific for *Apicystis bombi*. Bumble bee colonies that test positive for Trypanosomatid or Microsporidian pathogens using general primers should be examined microscopically and with species-specific primers in subsequent PCR reactions to verify the causative agent. Colonies should be selected randomly from throughout the rearing facility with colonies of various ages being inspected weekly. A stratified sampling scheme should be employed to select equal numbers of colonies that are two weeks from shipment, and a month from shipment, etc. Colony-level tests should include one non-callow worker or male bumble bee from each colony designated for testing each week. Broad testing of many colonies is desired for the random tests, but in-depth testing of colonies with disease symptoms is covered below. When a positive test result occurs, tests on individual colony samples should proceed within 24 hours and colonies with individual positive results should be moved to isolation until destroyed. If an outbreak is detected, testing frequency and intensity should be intensified in spatially and/or lineage-associated colonies for a one-month period. The random stratified testing should be supplemented with a pre-sale test of all colonies one week prior to shipping. A pooled testing strategy could be used to test for these pathogens and would allow for this high coverage sampling with low costs.

Not all bumble bee pathogens generate symptoms that can be detected easily visually, and some endosymbionts may not generate any symptoms in bumble bees. Thus the optimal testing strategy would be to sample individuals from every colony that is shipped from commercial production facilities. This level of sampling would ensure that no infected colonies would be shipped, thereby eliminating the potential risk for wild and managed pollinators.

### Quarantine and isolation

Any new stock brought into the facility should undergo a period of quarantine and testing before integration of that stock into the rearing operation. New queen stock should be kept isolated from main production lines until a full cycle of offspring is produced, observed for two weeks for symptoms, and tested for the primary disease agents using PCR detection protocols. Longer periods of isolation would increase confidence in disease-free status, but may not be necessary. New colonies that are brought into rearing facilities should first be tested with PCR by subsampling 5% of adult bees and then be observed for two weeks and retested before integration into main production facilities.

For main production and breeding lines in production a colony with any disease symptoms should immediately be isolated from other colonies and tested for known pathologies. Ten workers (a compromise between missing infected bees and significantly reducing colony size) from colonies with symptoms should be used for detection of known pathogens of concern. Recognizing that abiotic factors can cause pathology, colonies isolated with symptoms could be returned to production assuming that the causative agent for symptoms is determined and two weeks elapse from the resolution of symptoms and detectable infection. Colonies or individuals that are placed in isolation that test negative for known pathogens should likewise be held for two weeks after resolution of symptoms to ensure that a novel pathogen is not involved.

Newer tools may be required to confirm the presence of some parasites and pathogens in colonies. For example, next-generation sequencing technology was found to detect some pathogens that were not identified by PCR (Bartolomé et al. 2021). Ion PGM sequencing detected species that were not found by classical protocols (either specific PCR amplification or amplification with broad-range primers plus Sanger sequencing). The newer sequencing technique detected pathogens never reported previously in bumble bees (*Crithidia acanthocephali* and a novel neogregarinorida species) (Bartolomé et al. 2021).

If workers from the colonies test positive for *Vairimorpha bombi*, *V. ceranae*, *Crithidia bombi*, *C. expoeki* or *Apicystis bombi*, they should not be sold and should be destroyed to prevent disease spread within the facility. Destruction by freezing is recommended and a sample of ten workers and a portion of the brood comb and pollen should be held frozen for forensic purposes (see below). Infected biological and related material should be kept isolated at all times and disposed of in accordance with local biohazard regulations. At a minimum, material should be contained in two sealed plastic bags, one inside the other, until incineration or fumigation.

### Sanitation and prevention

Reducing pathogen levels in facilities should be accomplished through production controls including facility construction and materials management. For example, wild bumble bees should be prohibited from entering a production facility using double entrance doors, screening of ventilation ducts and maintaining positive air pressure in the buildings. Bees that are brought in intentionally to augment production or breeding stock should be processed through a self-imposed company quarantine. These bees must remain separate from production colonies until health can be verified. Equipment and shelving in facilities should be constructed of material that can be easily cleaned and sterilized (e.g., stainless steel, aluminum, etc.) or is disposable. Construction is ideally concrete and steel with floor drains for ease in cleaning. Colonies in production should be housed in plastic boxes that are either new, shipped from the facility at sale, or that have been cleaned and sterilized. Other nesting materials (cotton, wax, pollen, etc.) should not be reused.

To reduce pathogen exposure from feeding, food sources should be carefully controlled. Carbohydrate sources are proprietary; however, we do not know of any production facility that utilizes unsterilized sugar sources for feeding. Unpasteurized honey should not be used, but rather mixtures of sucrose, glucose, and fructose may be manufactured to optimize production and antimicrobial preservatives added; any additives should be openly reported. In addition to sterile sugar sources, some companies choose to sterilize pollen before it enters the facility. To date, gamma irradiation has been shown to reduce the viability of pathogens in pollen (Graystock et al. 2016) and does not severely reduce nutritive value (Yook et al. 1998), and any sterilization method that significantly reduces pathogen loads in bee feed would be a best management practice under a clean stock program. Although completely artificial protein diets are not yet available, pollen substitutes can reduce the use of pollen (Bortolotti et al. 2020), and possibly in the future they will replace pollen and eliminate the risks it poses. Equipment used for feeding should either not be shared among colonies or cleaned and sterilized between use in individual colonies. Processes that minimize the need for moving equipment between colonies are optimal.

Currently, best disease management and pest control strategies involve control of outbreaks in rearing facilities. Primary importance should be focused on rapid identification of disease outbreaks, proper disposal of infected hives and thorough equipment cleaning practices to reduce disease transmission between colonies (Huang et al. 2015). Hand sanitation of facility workers moving from colony to colony is also necessary to reduce pathogen transmission.

### Treatments

Treatment is generally implemented when prevention fails. Currently, very few treatments are readily available for the control and management of pathogens and pests within bumble bee colonies. Therefore, at this time we recommend destroying any infected colonies to reduce the likelihood of an outbreak in the rearing facility and wherever the bees are shipped. However, we provide a list of known or potential preventative measures and possible treatments as these may become useful tools in the future.

Monitoring and treatment methods exist for insect pests that infest rearing facilities. *Bacillus thuringiensis* can be used to control for wax moths (*Galleria mellonela* and *Achroia grisella*) (Burges & Bailey 1968) and Indian meal moths (*Plodia interpunctella*) (McGaughey 1976). Ultraviolet light traps can also be used for moth control. Bait and pheromone traps also exist for monitoring and controlling wax moths and Indian meal moths. Fruit flies can also be a nuisance in bumble bee rearing facilities; fly paper and bait traps are used to control them as outbreaks occur.

Chemical and biological controls of microbial pathogens in rearing facilities are not well developed. While fumagillin is used as a treatment for *Vairimorpha apis* in honey bees, it is not effective against *V. bombi* in bumble bee colonies (Whittington & Winston 2003); thus, sanitation of equipment and isolation of infected colonies is necessary when *V. bombi* is detected in rearing facilities. Solutions of sodium hypochlorite or ammonia have been shown to eliminate viable *V. ceranae* spores from surfaces (Rodríguez-García et al. 2022), and could also be effective against other Microsporidans such as *V. bombi*. Although sugar concentrations such as those found in nectars (Folly et al. 2020), and secondary compounds from nectar such as caffeine (Folly et al. 2021), can have negative effects on *V. bombi*, the potential for this to be used in commercial colonies has not been explored.

Research has revealed several promising treatments for potential development. Several studies have found that secondary metabolites found in nectar (particularly alkaloids) can help reduce parasite loads (specifically *C. bombi*) in bumble bees (Koch et al. 2022) and nectar containing alkaloids is preferentially chosen by bees if they are infected (Baracchi et al. 2015; Biller et al. 2015; Manson et al. 2010; Richardson et al. 2015). These studies have also demonstrated that secondary metabolites can present some negative side effects to the bees, as well. Moreover, there is growing evidence that sunflower pollen consistently reduces *C. bombi* infections in *B. impatiens*, both in the lab and in the field (Fowler et al. 2022a; Fowler et al. 2022b; Fowler et al. 2020; Giacomini et al. 2018; LoCascio et al. 2019), further highlighting the potential medicinal properties of different pollen types. Antiparasitic effects of natural plant compounds have attracted recent attention (Fitch et al. 2022; Koch et al. 2019), and further testing of these treatments is needed to determine their effectiveness as medicine for bumble bee colonies.

### Forensic and reporting capacity

Forensic capacity has several components, including the ability to identify the causative agents of disease, the sources of outbreaks, and tracing of contact of contagious individuals with healthy bees. Because disease can present after the colonies have shipped from rearing facilities, tracking records should be maintained with unique colony identifiers for two years after shipment. Rearing operations should maintain a database of these shipments including date shipped, destination, shipping origins and inspection data, and other information that could assist in tracing a disease outbreak to its origin.

Samples of diseased material from rearing facilities should be kept frozen at −20°C for a period of two years after the colony is destroyed. As described above, a sample of 10 bees (if available) and a portion of the brood comb and pollen from diseased colonies should be stored frozen and made available to research and regulatory groups requesting access. The remaining material from diseased nests should be contained in two sealed plastic bags before being destroyed, preferably through incineration or fumigation.

We recommend that a clean stock program includes a database of shipment information that is maintained in-house by commercial producers and made accessible to federal, provincial, and state regulatory agencies (*e.g*., USDA-APHIS, CFIA). Ideally, the database would be tri-national and include Canada, Mexico and the USA and could provide summary data on colony production numbers and shipment destinations to the public upon request. Data should be available quickly, protect the privacy of the production companies and end users, and be detailed enough to address problems as they arise.

## Concluding recommendations

This paper is, in part, a response to the current lack of knowledge of industry standards among the broader bee conservation and scientific community that has arisen from past reticence to share production and shipping details. We emphasize the urgent need to align bumble bee commercial practices with pollinator health goals stated in the USA’s National Strategy on Pollinators in the Pollinator Research Action Plan (2015), and biosecurity goals stated in the National Strategy for Biosurveillance (2012). Previous declines in wild bumble bee populations in North America occurred after commercial disease outbreaks, and *Vairimorpha bombi* has been linked to those declines. Whether these links are warranted or not, it is critical to build trust in the system among producers, end users, and the conservation community. Adoption of a clean stock program, based on the best available science, adaptable to new threats, responsive to changes in data, and with greater information flow to conservation organizations, would be a strong step to recovering trust among communities and toward meeting the goals for agricultural biosecurity outlined in the National Strategy for Biosurveillance (2012).

We acknowledge that the implementation of a clean stock program could occur at many levels: municipal, state, federal, or international; however, it is most likely and perhaps most manageable for industry standards to be adopted with a third-party oversight or certification of the process, such as the dairy certifications by the Farmers Assuring Responsible Management program. Whereas many agriculturally produced products including plant material (e.g., Certified Seed programs, Federal Seed Act) and livestock (e.g., Animal Disease Traceability) are subject to federal regulations, the production, sale, and transport of pollinators has largely avoided regulation in North America. Currently in the United States, only Oregon and California have regulations governing bumble bee importation. While movement of bees across international borders allows for certain regulatory requirements, there is not a unified set of state regulations in the United States or among Canadian provinces, so industry standards could alleviate calls for additional regulation at either the state or federal level. In addition to adopting disease and pest control measures, reporting of sales and distribution numbers on a state-by-state (province-by-province) basis would allow regulators, wildlife managers, and scientists to respond appropriately to disease outbreaks in wild populations around commercial facilities. Additionally, a clean stock program could help ensure the future health of commercial bumble bee populations in rearing facilities and avoid situations such as the collapse of commercial *Bombus occidentalis* populations in the late 1990s. The assurances of clean stock to conservation organizations and government wildlife managers are especially critical to states and provinces with declining bumble bee populations, such as *B. affinis*, the Rusty Patched Bumble Bee, where Endangered Species Act protections might invoke a formal assessment and restrictions on bumble bee sales in the absence of verifiable production and trade data.

## Priority action recommendations:

A meeting of commercial bumble bee production representatives, bee conservation community partners, bumble bee scientists, and agency representatives in Canada, Mexico, and the United States of America to discuss common interests and needs and to develop components of a bumble bee movement program including clean stock certification and oversight. This meeting should focus on:

- Mitigating impacts to federally listed at-risk species, including identifying potentially deleterious pathogens and parasites
- Standards of clean stock certification program
- Shipment reporting and tracking
- Program management, implementation, and oversight
